# Can the SARS-CoV-2 Spike Protein Bind Integrins Independent of the RGD Sequence?

**DOI:** 10.3389/fcimb.2021.765300

**Published:** 2021-11-18

**Authors:** Christopher A. Beaudoin, Samir W. Hamaia, Christopher L.-H. Huang, Tom L. Blundell, Antony P. Jackson

**Affiliations:** ^1^Department of Biochemistry, Sanger Building, University of Cambridge, Cambridge, United Kingdom; ^2^Department of Biochemistry, Hopkins Building, University of Cambridge, Cambridge, United Kingdom; ^3^Physiological Laboratory, University of Cambridge, Cambridge, United Kingdom

**Keywords:** SARS-CoV-2, SARS-CoV-2 spike protein, integrin, integrin-binding motif, RGD, bioinformatics

## Abstract

The RGD motif in the Severe Acute Syndrome Coronavirus 2 (SARS-CoV-2) spike protein has been predicted to bind RGD-recognizing integrins. Recent studies have shown that the spike protein does, indeed, interact with α_V_β_3_ and α_5_β_1_ integrins, both of which bind to RGD-containing ligands. However, computational studies have suggested that binding between the spike RGD motif and integrins is not favourable, even when unfolding occurs after conformational changes induced by binding to the canonical host entry receptor, angiotensin-converting enzyme 2 (ACE2). Furthermore, non-RGD-binding integrins, such as α_x_, have been suggested to interact with the SARS-CoV-2 spike protein. Other viral pathogens, such as rotaviruses, have been recorded to bind integrins in an RGD-independent manner to initiate host cell entry. Thus, in order to consider the potential for the SARS-CoV-2 spike protein to bind integrins independent of the RGD sequence, we investigate several factors related to the involvement of integrins in SARS-CoV-2 infection. First, we review changes in integrin expression during SARS-CoV-2 infection to identify which integrins might be of interest. Then, all known non-RGD integrin-binding motifs are collected and mapped to the spike protein receptor-binding domain and analyzed for their 3D availability. Several integrin-binding motifs are shown to exhibit high sequence similarity with solvent accessible regions of the spike receptor-binding domain. Comparisons of these motifs with other betacoronavirus spike proteins, such as SARS-CoV and RaTG13, reveal that some have recently evolved while others are more conserved throughout phylogenetically similar betacoronaviruses. Interestingly, all of the potential integrin-binding motifs, including the RGD sequence, are conserved in one of the known pangolin coronavirus strains. Of note, the most recently recorded mutations in the spike protein receptor-binding domain were found outside of the putative integrin-binding sequences, although several mutations formed inside and close to one motif, in particular, may potentially enhance binding. These data suggest that the SARS-CoV-2 spike protein may interact with integrins independent of the RGD sequence and may help further explain how SARS-CoV-2 and other viruses can evolve to bind to integrins.

## Introduction

Integrins are an ancient superfamily of heterodimeric transmembrane proteins that are involved in diverse cell processes, such as cell-cell adhesion and both inside-out and outside-in cell signaling, at cell surfaces (Sebé-Pedrós et al., 2010)⁠. The heterodimers consist of an alpha (α) and a beta (β) subunit, of which there are as many as 18 α and eight β subunits that form at least 24 heterodimeric structures known in humans (Takada et al., 2007). Some integrins, such as α_V_β_3_, are ubiquitously expressed across human tissues, while others are specific to certain cell types, such as α_IIb_β_3_ to platelets (Etzioni, 1999)⁠. In total, integrins recognize a large variety of cell surface, extracellular matrix, and soluble ligands (Takada et al., 2007). The most frequently observed integrin-binding motif, RGD, is present on several extracellular proteins, such as fibronectin and vitronectin, and can interact with at least 12 integrin subtypes (Ruoslahti, 1996)⁠. This motif uses the negatively-charged aspartate to interact with positively-charged cations in the metal ion–dependent adhesion site (MIDAS) domain of integrins (Xiong et al., 2002)⁠. Other non-RGD motifs have been discovered to be integrin-binding, such as the LDV sequence from vascular cell adhesion molecule and the GLOGER sequence (where O is hydroxyproline) from collagen that bind α_4_ and α_1_β_1_ integrins, respectively (Makarem and Humphries, 1991; Barczyk et al., 2010)⁠. Designed *de novo* peptides and peptides homologous to natural ligands, such as ATN-161 (Ac-PHSCN-NH2), have also been described to recognize integrins and some do not share sequence identity or similarity to known integrin-binding motifs (van Golen et al., 2002; Cianfrocca et al., 2006). Thus, integrin-ligand specificity is still incompletely understood and may involve interactions with varying motifs that correspond to binding sites on different domains of integrin dimers (Luo et al., 2007).

Because integrins are largely accessible on the surface of human cells, they are a common target for cell entry by viral pathogens (Maginnis, 2018)⁠. Surface proteins of some viruses, such as the capsid protein VP1 of coxsackievirus A9, contain commonly known integrin-binding sequences, such as RGD, that bind integrins to gain entry into cells or modulate downstream signaling pathways, while other viruses have acquired novel integrin-binding mechanisms, for example the binding of human echovirus 1 outside of the known ligand-binding site on α_2_β_1_ integrin (Roivainen et al., 1994; Jokinen et al., 2010; Hussein et al., 2015)⁠. Other viruses are known to bind integrins, such as HIV-1 binding to α_4_β_7_, but the specific residues involved in the interactions are still unknown (Liu and Lusso, 2020)⁠. Thus, further investigation of the evolution of integrin-binding for viral pathogens is important for a better understanding of viral cell entry and cell signaling perturbations during infection.

The Severe Acute Syndrome Coronavirus 2 (SARS-CoV-2) spike protein receptor-binding domain, which binds to angiotensin-converting enzyme 2 (ACE2) to initiate viral cell entry, has been shown to contain an exposed RGD motif that has been suggested to be integrin-binding, thus potentially allowing for integrin-mediated cell entry (Luan et al., 2020; Makowski et al., 2021). Experimental studies have shown that the SARS-CoV-2 spike protein directly binds α_V_β_3_ and α_5_β_1_ integrins and may also interact with α_3_, β_1_, α_4_, and α_X_ integrin subunits (Aguirre et al., 2020; Kotani and Nakano, 2020; Beddingfield et al., 2021; Nader et al., 2021; Wang et al., 2021). RGD-based drugs were found to inhibit spike binding to α_V_β_3_ and α_5_β_1_ integrins, and mutating the RGD motif to RGE or RGA was also found to decrease binding to α_V_β_5_ integrins – further implicating the spike RGD motif in interactions with integrins (Sebé-Pedrós et al., 2010; Robles et al., 2021)⁠. Additionally, the canonical SARS-CoV-2 cell entry receptor, ACE2, has been shown to associate with α_5_β_1_ integrins, potentially facilitating virus internalization (Beddingfield et al., 2021; Kliche et al., 2021)⁠. A structure-based computational study has indicated that the RGD motif accessibility to integrins depends based on its depth within the spike receptor-binding domain (Othman et al., 2021). The study performed protein-protein docking between the spike protein and α_5_β_1_, α_IIb_β_3_, and α_V_β_8_ integrins and found that the interactions were not favourable, thus suggesting that other motifs on the spike receptor-binding domain may be involved in interactions with integrins. Also, some of the integrins discovered to be associated with SARS-CoV-2 cell entry, such as α_X_, are not known to bind RGD peptides (Wang et al., 2021)⁠. No definitive structural study has, yet, been described for spike-integrin interactions. Thus, although the RGD sequence has been shown as the probable integrin-binding motif for RGD-binding integrins, the jury is still out on motifs on the spike protein that allow binding to non-RGD-binding integrins or if other spike motifs synergize with RGD-related binding to integrins.

The presence of non-RGD integrin-binding motifs on coronavirus, particularly SARS-CoV-2, spike proteins has not been systematically investigated. Herein, to infer potential connections, we investigate integrins that have been demonstrated to be directly involved in binding to the spike protein, that have been shown to mediate viral entry through the spike protein, or that exhibit changed expression over the course of SARS-CoV-2 infection. Furthermore, we have utilized sequence and structure-based bioinformatics approaches to improve our understanding of the potential of spike binding to integrins by cataloguing all known integrin-binding motifs and mapping them to the SARS-CoV-2 spike protein receptor-binding domain. Since the N-terminal receptor-binding domain of the SARS-CoV-2 spike protein is heavily glycosylated, the C-terminal receptor-binding domain – which contains the main interaction site with ACE2 – was chosen for study. Amino acid sequence alignments revealed that some exact and several similar matches were discovered between known integrin-binding motifs and the SARS-CoV-2 spike protein. Subsequent structural analyses reveal that the three-dimensional availability of some motifs is greater than that of the RGD sequence. The evolution of these motifs from phylogenetically close betacoronaviruses, such as SARS-CoV and RaTG13, was also analyzed alongside recently recorded mutations in the SARS-CoV-2 spike protein. The combination of these ideas suggests that integrin binding may also occur independently of the RGD motif on the spike protein. Further experimental work is necessary to validate potential spike-integrin interactions.

## Integrins and SARS-CoV-2

Within the extensive research into SARS-CoV-2, several studies have outlined changes in integrin expression and some have shown direct interactions between the spike protein and different integrins. Since increases in integrin expression may indicate their potential usage as cell entry receptors by the spike protein, as previously described with dengue virus serotype 2 and β_3_ integrins, a meta-analysis of expressed integrins during SARS-CoV-2 infection may give insight into which corresponding integrin-binding motifs may be of interest (Zhang et al., 2007; Calver et al., 2021; Gisby et al., 2021)⁠.

Binding assays have shown that the spike protein directly interacts with α_5_β_1_ and α_V_β_3_ integrins (Beddingfield et al., 2021; Nader et al., 2021)⁠. One study discovered that the ATN-161 pentapeptide inhibited interactions between integrin α_5_β_1_ and the SARS-CoV-2 spike protein, for which they predicted three inhibitory ATN-161 binding sites on α_5_β_1_ (Beddingfield et al., 2021). Cilengitide, an RGD-based drug, and an RGD peptide were found to block the SARS-CoV-2 spike protein from binding to α_V_β_3_ and α_5_β_1_ integrins, respectively, indicating that the spike protein binds in an RGD-dependent manner (Nader et al., 2021; Robles et al., 2021). A co-immunoprecipitation study found that mutating the RGD sequence to RGE or RGA reduced the binding of the SARS-CoV-2 spike protein to α_V_β_5_ integrins (Gao et al., 2021)⁠. Binding assays demonstrated direct binding between the spike protein and β_1_ integrins (Park et al., 2021)⁠. A screening of candidate cell entry receptors for SARS-CoV-2 suggested that α_3_ and β_1_ integrins may be involved, and a CRISPR activation screening looking at neuronal receptors involved in SARS-CoV-2 infection found that α_X_ integrins may mediate cell entry (Kotani and Nakano, 2020; Wang et al., 2021). The antibody natalizumab, which binds to α_4_ integrins, was found to decrease SARS-CoV-2 infection, hinting at the potential use of these integrins in cell entry (Aguirre et al., 2020)⁠. These studies provide more definitive proof that integrins interact with the SARS-CoV-2 spike protein.

Reviewing changes in integrin expression during SARS-CoV-2 infection may give clues about their potential roles as viral cell entry receptors: increases in expression suggest potential usage, while decreases in expression may rule out their involvement (Fantini and Yahi, 2015). Notably, however, integrin expression may also be an indicator of several other altered cellular processes, such as inflammation and apoptosis, during infection (Futosi et al., 2013)⁠. Furthermore, increased integrin expression has also been shown to enhance viral replication irrespective of binding to viral surface proteins, and decreased expression may result as a viral mechanism to prevent multiple viruses from infecting the same cell or as a defence mechanism by the host (Michel et al., 2005; Lanier, 2008; Schmidt et al., 2013)⁠. Integrins that showed increased expression were selected since expression of the canonical SARS-CoV-2 entry receptor, ACE2, has also been shown to increase following infection (Zamorano Cuervo and Grandvaux, 2020)⁠. Three publicly-available expression datasets from the Gene Expression Atlas collectively showed that there were increases in α_1_, α_V_, and β_1_ integrin expression and decreases in α_3_, α_4_, α_D_, β_4_, and β_7_ integrin expression post SARS-CoV-2 infection in colon cells and increases in α_2_, α_7_, α_M_, α_V_, β_2_, and β_8_ integrin expression and decreases in α_D_ and β_7_ integrin expression in infected lung cells (**Supplementary Table 1**) (Desai et al., 2020; Vanderheiden et al., 2020; Wu et al., 2020)⁠. One study looked at longitudinal proteomic profiles in SARS-CoV-2 patients and found that the presence of β_6_ integrins increased over time while α_11_ integrins decreased (Gisby et al., 2021). A mass cytometry screening of transmembrane protein expression of platelets from SARS-CoV-2 patients found increased expression of α_IIb_β_3_ integrins (Bongiovanni et al., 2021). These studies suggest that the expression patterns of several integrin subtypes are influenced by SARS-CoV-2 infection and that several are available as potential receptors.

Altogether, the data suggest that α_1_, α_2_, α_4_, α_7_, α_M_, α_V_, α_X_, β_1_, β_2_, β_6_, β_8_, α_5_β_1_, α_V_β_3_, and α_IIb_β_3_ integrins could be of use to the SARS-CoV-2 spike protein. The α_2_, α_4_, α_7_, α_V_, β_1_, β_6_, β_8_, α_5_β_1_, α_V_β_3_, and α_IIb_β_3_ integrins are all known to be RGD-binding (or part of dimers that bind RGD for individual alpha or beta subunits), while the α_1_, α_M_, α_X_, and β_2_ are not known to be part of dimeric complexes that bind RGD sequences, which suggests that spike could potentially interact with integrins using motifs outside of the RGD sequence. Of note, the expression and direct experimental studies have been limited by cell type, and other integrin subtypes, such as α_E_ and α_L_, have not been extensively explored. Thus, an evaluation of potential non-RGD integrin-binding motifs present on the SARS-CoV-2 spike protein may help guide further structural investigations and may shed light on the integrin-binding potential of other viruses.

## Non-RGD Potential Integrin-Binding Motifs on the SARS-CoV-2 Spike Protein Receptor-Binding Domain

The use of non-RGD integrin-binding motifs on the SARS-CoV-2 spike protein has only been explored briefly: an LDI motif was discovered on the outside of the C-terminal receptor-binding domain (RBD) (Tresoldi et al., 2020)⁠. Thus, to expand the understanding of potential non-RGD integrin-binding sites on the SARS-CoV-2 protein, we gathered all known non-RGD integrin-binding motifs and compared them to the sequence of the SARS-CoV-2 RBD and further analyzed their structural accessibility for potential binding to integrins. The RGD motif was analyzed in parallel as a reference.

To collect all known integrin-binding motifs, we searched the PepBank and ELM web servers using “integrin” and “integrin-binding” search terms and selected all non-RGD motifs that were returned (Shtatland et al., 2007; Duchrow et al., 2009; Kumar et al., 2020)⁠. We additionally performed a literature search to include motifs from viruses and *de novo* or homology-derived peptides not found in the database queries. The search resulted in a total of 71 motifs found from ELM (6), PepBank (36), and the literature search (29) (**Supplementary Table 2**).

The motifs were aligned to the sequence of the SARS-CoV-2 spike receptor-binding domain (residues 333-523) using EMBOSS Needle and the EBLOSUM62 similarity matrix and sequence identity and similarity were recorded (Madeira et al., 2019)⁠. The reverse sequence of the integrin-binding motifs was also screened against the RBD sequence, since DGR sequences have been found to bind RGD-recognizing integrins (Spitaleri et al., 2008). We focused on motifs that reported amino acid similarity percentages above 50%, although manual inspection of the alignments revealed some alignments that had similar but out of order amino acids, which may still exhibit similar physicochemical characteristics.

**Figure 1:**
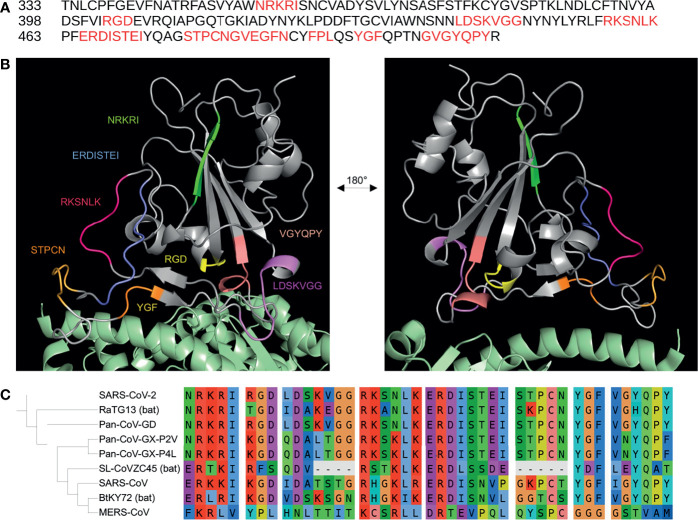
Putative integrin-binding motifs on the SARS-CoV-2 receptor-binding domain.The SARS-CoV-2 receptor-binding domain (RBD) amino acid sequence is shown with the putative integrin-binding motifs marked in red **(A)**. The SARS-CoV-2 spike RBD is depicted with the predicted structurally-accessible integrin-binding motifs highlighted in different colors **(B)**. The spike RBD is shown aligned with the PDB: 6m0j structure in order to show the potential accessibility of these motifs when the spike protein is bound to the canonical host cell entry receptor ACE2 (green) (B). A sequence alignment of the putative integrin-binding motifs found on the SARS-CoV-2 receptor binding domain with spike sequences from the SARS-CoV, RatG13, MERS-CoV, Pan-CoV-GD, Pan-CoV-GX-P4L, Pan-CoV-GX-P2V, SL-CoVZC45, BtKY72 betacoronavirus strains is shown with a corresponding phylogenetic tree of the coronavirus genomes generated using FastTree 2.1 and visualized with iTOL **(C)**.

The sequence mapping resulted in 27 integrin-binding motifs that align to 11 motifs on the SARS-CoV-2 spike RBD (**Table 1** and **Figure 1A**). Except for RGD, only one motif, the LDS sequence that binds α_4_β_1_ and α_5_β_1_ integrins, on the SARS-CoV-2 spike RBD was found to match at 100% identity in the forward direction; however, one motif read in the reverse direction, TEI, was found to align at 100% identity to the [L,I]ET motif that has been shown to bind α_L_β_2_ integrins. Using amino acid similarity, the spike RKSNLK motif aligned at 100% similarity to the α_3_β_1_-binding N[G,V]R and α_V_β_6_-binding QRSDL motifs. In the reverse direction, the α_IIb_β_3_-binding VPW motif and the YGL motif, which binds α_4_β_1_, α_4_β_7_, α_9_β_1_ integrins, were found to be 100% similar to the spike FPL and VGY motifs, respectively. Several α_V_β_6_-binding motifs – RTDLY, REDV, ASDIS, RTDLS, RDLET – were found to be between 50.0% and 66.6% similar to the ERDIS motif on the spike protein, which just precedes the TEI sequence. The spike MLD motif, which binds α_4_β_1_, α_9_β_1_, α_7_β_4_ integrins, was also found to have 66.6% identity to the aforementioned LDS motif. The α_1_β_1_-binding (R/K)TS motif was found to be 66.6% similar to the previously mentioned RKSNLK motif. The α_V_β_3_-binding GRKRK and GRFPF motifs were found to be 60% similar to the NRKRI and GYQPY (overlapping the previously mentioned VGY motif) spike motifs. The LLG motif, which has been shown to bind β_2_ integrins, was found to be 60% similar to both the VGG and GVG motifs on the spike RBD. Interestingly, the ATN-161 pentapeptide (Ac-PHSCN-NH(2)) was discovered to be 42.9% identical to the spike RBD STPCN motif, while the α_X_β_2_-binding GPRP motif also aligned at 50% similarity to the overlapping GSTP spike motif. The G[F,L,V][P,O]GE[N,R] motifs, which have been shown to bind α_1_β_1_ and α_2_β_1_ integrins, were found to be ~ 50% similar to the spike GVEGFN motif. The spike YGF motif was found to be 66.6% similar to the YGL motif. Altogether, these motifs comprise the potential for the spike protein to bind 12 integrin subtypes independent of the RGD sequence.

**Table 1:** Known integrin-binding motifs mapped to the SARS-CoV-2 receptor-binding domain (RBD).

SARS-CoV-2 RBD Residues	SARS-CoV-2 Motif	Integrin-binding Motif	% Identity/% Similarity (reverse AA order)	Binding integrin(s) (weak binding)
403-405	RGD	RGD	100/100	α_5_β_1_,α_8_β_1_,α_V_β_1_,α_V_β_3_, α_V_β_5_, α_V_β_6_, α_V_β_8_, α_IIb_β_3_, (α_2_β_1_), (α_3_β_1_), (α_4_β_1_), (α_4_β_7_), (α_7_β_1_)
354-358	NRKRI	GRKRK	60/60	α_V_β_3_
444-447/502-504	KVGG/GVG	LLG	33.3/66.6	β_2_
457-462	RKSNLK	N[G,V]R	33.3/100	α_3_β_1_
		(R/K)TS	66.6/66.6	α_1_β_1_
		QRSDL	40/100	α_V_β_6_
466-472	ERDISTEI	[L,I]ET	33.3/33.3 (100/100)	α_L_β_2_
		RTDLY	33.3/66.6	α_V_β_6_
		REDV	25/50	α_V_β_6_
		ASDIS	60/60	α_V_β_6_
		RTDLS	60/66.6	α_V_β_6_
		RDLET	33.3/66.6	α_V_β_6_
476-482	GSTPCN	PHSCN	42.9/42.9	α_5_β_1_,α_V_β_1_,α_V_β_3_
		GPRP	50/50	α_X_β_2_
490-492	FPL	YGL	33.3/66.6	α_4_β_1_,α_4_β_7_,α_9_β_1_
		VPW	33.3/33.3 (33.3/100)	α_IIb_β_3_
503-505	VGY	YGL	33.3/33.3 (66.6/100)	α_4_β_1_,α_4_β_7_,α_9_β_1_
		VPW	33.3/66.6	α_IIb_β_3_
SARS-CoV-2 motifs excluded based on structural and functional analyses				
**SARS-CoV-2 RBD Residues**	**SARS-CoV-2 Motif**	**Integrin-binding Motif**	**% Identity/% Similarity (reverse AA order)**	**Binding integrin(s) (weak binding)**
441-445	LDS	IDS/LDV	100/100	α_4_β_1_,(α_5_β_1_)
		MLD	66.6/66.6	α_4_β_1_,α_9_β_1_,α_7_β_4_
482-487	GVEGFN	GFPGER/GLOGEN	50/50	α_1_β_1_,α_2_β_1_
496-498	YGF	YGL	66.6/66.6	α_4_β_1_,α_4_β_7_,α_9_β_1_
504-508	GYQPY	GRFPF	40/60	α_V_β_3_

The presence of motifs on the SARS-CoV-2 spike protein that are 100% identical or similar to integrin-binding motifs brings attention to the potential for binding to integrins independent of the RGD sequence. Some of the spike motifs, such as GVG and VGY, are overlapping, which may induce stronger binding or permit the binding of different integrins on one overarching motif. Interestingly, some spike motifs were highly similar to integrin-binding motifs but the amino acids were out of order. For example, the spike ERDI motif was found to be 50% similar to the α_V_β_6_-binding REDV motif by alignment of the D and I/V residues, but the E and R residues were found on both motifs in a different order. This was also the case for the similarity between the ATN-161 pentapeptide sequence (PHSCN) and the spike STPCN motif, in which both the P and S residues are present but in a different order. Furthermore, the similarity to the ATN-161 pentapeptide is interesting considering studies that suggest its protective ability against SARS-CoV-2 infection, although the peptide has been suggested to also bind the RGD-binding site on integrins (Amruta et al., 2021; Beddingfield et al., 2021)⁠. Structural analysis to investigate the accessibility of the motifs on the spike protein may lend more insights into their potential ability to bind integrins.

## Structural Orientation of Potential Integrin-Binding Motifs

The alignments and the amino acid configurations of the corresponding spike protein motifs were subsequently inspected on the structure of the spike protein RBD [3D model generated as previously described (Beaudoin et al., 2021)] to determine whether the side chains are found on the surface of the receptor-binding domain. Since the literature on the 3D structure of binding sites for non-RGD motifs is limited and the potential binding modes may be numerous, in order to quantitatively assess motif accessibility, structural flexibility and solvent accessibility were determined to better understand the surrounding residue microenvironment. Ten 3D models representing different points in flexibility for the SARS-CoV-2 RBD were generated using CABS-Flex 2.0, which utilizes molecular dynamics to infer root mean squared flexibility (RMSF) for each residue, and the solvent accessibility (defined as the number of water molecules in contact with the residue * 10) for each residue in each model was calculated using DSSP (**Table 2**; **Supplementary Table 3**) (Kabsch and Sander, 1983; Kuriata et al., 2018). Overall, as discussed in several studies, the SARS-CoV-2 RBD does not exhibit extensive flexibility, but the distal ends of the ACE2 binding motif in particular, which are often unresolved in crystal structures, were reported with the highest flexibility (~ 6 RMSF) (Dehury et al., 2020; Spinello et al., 2020). The mean RMSF for all residues of interest was 1.84, and the average solvent accessibility was 65.70.

**Table 2:** Root mean square flexibility (RMSF) and average solvent accessibility (SA) values for the putative integrin-binding motifs on the SARS-CoV-2 spike protein receptor-binding domain.

Residue #	Residue	RMSF	Average SA
354	N	0.31	63.18
355	R	0.50	119.18
356	K	0.46	62.27
357	R	0.43	162.45
358	I	0.66	6.82
	Average	0.47	82.78
403	R	0.24	49.09
404	G	0.50	4.18
405	D	1.02	87.27
	Average	0.59	46.85
441	L	1.15	74.00
442	D	0.90	3.18
443	S	1.06	14.64
	Average	1.04	30.61
444	K	1.42	154.45
445	V	2.44	61.55
446	G	2.68	42.73
447	G	1.72	46.45
	Average	2.06	76.30
457	R	0.92	110.73
458	K	1.40	46.91
459	S	1.79	59.45
460	N	1.77	89.82
461	L	1.70	17.00
462	K	2.04	106.36
	Average	1.60	71.71
465	E	1.37	73.64
466	R	0.72	65.09
467	D	1.61	77.64
468	I	1.05	89.82
469	S	1.18	32.27
470	T	1.20	83.55
471	E	1.12	58.45
472	I	1.58	82.82
	Average	1.23	70.41
477	S	3.78	66.45
478	T	3.46	32.09
479	P	4.31	67.09
480	C	5.21	85.73
481	N	5.38	110.45
	Average	4.43	72.36
482	G	5.92	57.36
483	V	5.48	91.36
484	E	5.24	131.27
485	G	4.54	59.82
486	F	3.38	122.55
487	N	2.61	94.27
	Average	4.48	82.57
490	F	0.75	75.09
491	P	0.57	39.64
492	L	0.40	74.82
	Average	0.57	63.18
495	Y	0.45	15.55
496	G	0.45	21.73
497	F	0.37	38.55
	Average	0.42	25.27
502	G	3.99	78.09
503	V	2.80	72.45
504	G	2.05	49.73
505	Y	0.85	103.45
506	Q	0.61	67.91
507	P	0.42	1.09
508	Y	0.21	15.91
	Average (GVG)	2.94	66.76
	Average (VGY)	1.90	75.21
	Average (VGYQPY)	0.83	47.62

After structural analysis, 19 integrin-binding motifs that cover 8 motifs on the spike RBD were found as potentially accessible to receptors, while 3 of the original 11 spike motifs were discarded due to their buriedness or incompatibility with potential binding to integrins based on the knowledge of the original integrin-motif interactions (**Figure 1B**). The STPCN and GVEGFN motifs, which are found adjacent to each other in sequence, exhibited the highest flexibility with an average of 4.43 and 4.48 RMSF, respectively, and high average solvent accessibility of 72.36 and 82.57, respectively. However, after noting that the GVEGFN motif is 1) missing a crucial glutamate between the glycine and asparagine residues and 2) that recent mutation E484K exchanges the remaining acidic glutamate for a basic lysine, this motif was deemed unlikely to mimic integrin-binding in the manner that has been shown for natural G[F,L,V][P,O]GE[N,R] ligands (Hamaia et al., 2012; Wise, 2021)⁠. Thus, GVEGFN was discarded. The KVGG motif reported the next highest combined flexibility and solvent accessibility at 2.06 RMSF and 76.29, respectively. The GVG motif, which sits directly in front of the RGD motif, also exhibited a high mean RMSF (2.94) and a solvent accessibility of 66.76. The RKSNLK and ERDISTEI reported average RMSF values of 1.60 and 1.23, respectively, and solvent accessibility values of 71.71 and 70.41, respectively. The overlapping GVG, VGY, and GYQPY motifs exhibited RMSF values of 2.94, 1.90, 0.83, respectively, and solvent accessibility values of 66.76, 75.21, 47.62. Because of the low flexibility and solvent accessibility of residues in the GYQPY motif, it was discarded as potentially integrin-binding. The LDS motif was found to exhibit low flexibility (1.04 RMSF) and the lowest average solvent accessibility of all putative motifs at 30.61. Because the aspartate residue, which is important for binding in the MIDAS region of integrins, in the LDS motif was buried inside the RBD (solvent accessibility of D: 3.53), the LDS motif was discarded (Ruoslahti, 1996). The FPL motif exhibited a low average flexibility at 0.571 RMSF but a mean solvent accessibility (63.18) close to the average of other motifs. The NRKRI motif reported the second-lowest average flexibility (0.47 RMSF) but the highest average solvent accessibility of 82.78. The YGF motif showed the lowest mean flexibility (0.42 RMSF) and solvent accessibility (25.27) and was, thus, discarded.

Although the RGD motif was described as inaccessible for integrin-binding by previous computational analyses, the R and D residues were found to exhibit low flexibility overall – 0.244 and 1.015 RMSF, respectively – but high average solvent accessibility – 49.09 and 87.27, respectively. The RGD motif reported an average 0.585 RMSF and a solvent accessibility of 46.85. The reference model showed low solvent accessibility for the arginine and aspartate residues (R: 24 and D: 27), which could imply that small conformational changes may have large effects on residue accessibility. Furthermore, the GVG motif in front of the RGD motif in 3D space showed high flexibility (2.94 RMSF), which may allow for restructuring to accommodate potential RGD-based interactions.

The resulting motifs comprise the potential binding to β_2_, α_1_β_1_, α_3_β_1_, α_4_β_1_, α_4_β_7_, α_9_β_1_, α_V_β_1_, α_V_β_3_, α_V_β_6_, α_L_β_2_, α_X_β_2_, and α_IIb_β_3_ integrins. Interestingly, several of these integrins overlap those that were suggested to directly interact with the spike protein or exhibit increased expression during SARS-CoV-2 infection: α_1_, α_4_ α_V_, α_X_, β_1_, β_2_, β_6_, α_V_β_3_, α_IIb_β_3_. Since many of the integrins overlap RGD-binding integrins, perhaps the putative non-RGD motifs on the spike protein may provide synergistic binding with the RGD sequence. The RGD motif is overall less accessible than some of the newly discovered potential integrin-binding motifs, such as STPCN and ERDISTEI, although the changes in solvent accessibility due to small conformational changes may have a significant impact on potential integrin-binding. Given that, however, several other motifs, such as the RKSNLK, VGY, and NRKRI, have strong potential for binding to integrins based on amino acid identity and similarity and 3D availability. Further experimental work is needed to validate these potential interactions.

## Evolution of Putative Non-RGD Integrin-Binding Motifs

As previously mentioned, the evolution of integrin-binding motifs on viral surface proteins provides another route for potential host cell entry. Thus, a look at the conservation of these motifs in related betacoronaviruses and an inspection of recently recorded mutations in the SARS-CoV-2 spike RBD may provide insights into the relevance of these motifs.

Recent genomic comparisons between the spike sequence of SARS-CoV-2 and other coronaviruses have shown that the receptor-binding domain is under both purifying and positive selection (Berrio et al., 2020; Li et al., 2020)⁠. Mutations have been suggested to increase affinity for ACE2 or to prevent recognition by neutralizing antibodies (Armijos-Jaramillo et al., 2020; Greaney et al., 2021). As shown in **Figure 1C**, an alignment of the SARS-CoV-2 spike receptor-binding domain sequence (NCBI Accession: NC_045512) with the sequences of the SARS-CoV (NC_004718); bat coronavirus strains RaTG13 (MN996532), SL-CoVZC45 (MG772933), and BtKY72 (KY352407); pangolin coronavirus strains GD (MT121216), GX-P4L (MT040333), and GX-P2V (MT072864); and the more phylogenetically distant MERS-CoV (NC_019843) shows that some of the motifs are completely conserved among recently diverged viruses while others have evolved more recently (Price et al., 2010; Letunic and Bork, 2021). Notably, only the pangolin GD strain was to share 100% amino acid sequence identity with all of the potential integrin-binding motifs, including the RGD sequence, from SARS-CoV-2 (Liu et al., 2020). The pangolin strain RGD sequence has not yet been mentioned in the literature, and its presence reveals that integrin-binding motifs may evolve before zoonotic transmission to humans. Some of the motifs, such as ERDISTEI and NRKRI, are completely conserved among the RaTG13 and pangolin coronavirus strains, while other motifs, such as RKSNLK and STPCN, seem to have evolved more recently. The alignments support studies that suggest that SARS-CoV-2 is a result of recombination between human, pangolin, and bat coronaviruses (Li et al., 2020)⁠. In addition to the analysis of the integrin-binding potential of these motifs, these insights can provide clues towards SARS-CoV-2 spike evolution.

Interestingly, the recent SARS-CoV-2 spike mutations seem to only directly affect the STPCN motif – where the G476S mutation adds another serine just before the serine already present and the S477N and T478K mutations affect the sequence itself (Guruprasad, 2021). Notably, the T478K mutation found in the Delta strain would create a more similar sequence to the aligned ATN-161 (PHSCN) sequence by replacing the polar threonine with a basic lysine, which may mimic basic histidine interactions (Aoki et al., 2021)⁠. The lack of mutations near or on the other putative integrin-binding sites, especially considering the conservation with the pangolin coronavirus genome, may give credibility to their integrin-binding potential.

The combination of 1) the conservation among recent betacoronaviruses and 2) the lack of mutations or insertion of mutations that provide better alignments for the putative integrin-binding motifs on the SARS-CoV-2 protein reveals that these sites may, indeed, be involved in interactions with integrins.

## Discussion

The SARS-CoV-2 spike protein has been shown to bind multiple integrin subtypes. Although there is growing evidence for interactions based on the spike RGD sequence, the exact binding mechanisms are still unknown (Robles et al., 2021; Simons et al., 2021)⁠. Additionally, previous preliminary computational studies suggest that the spike RGD motif is not completely accessible for integrin-binding, while there have also been other non-RGD-binding integrins that have been suggested to be potentially involved in SARS-CoV-2 viral entry (Othman et al., 2021; Wang et al., 2021). An analysis of putative non-RGD integrin-binding motifs on the SARS-CoV-2 receptor-binding domain identified several viable candidates after sequence alignment and subsequent structural characterization. The motifs comprise potential binding to RGD-binding integrins, such as α_V_β_6_, as well as non-RGD-binding integrins, such as α_X_β_2_. A meta-analysis of reported integrin expression levels revealed that several of the putative spike-binding partners are increased during SARS-CoV-2 infection, further adding to their validity. Since α_X_ integrins were both found as putative cell entry receptors for the SARS-CoV-2 spike protein in a CRISPR activation screen, the results presented in this study may help unravel potential binding mechanisms (Wang et al., 2021)⁠. The identified motifs may act together with the RGD motif or independently to bind integrins for cell entry or to interfere with cell signaling pathways.

Of interest, one motif was found to be similar to the ATN-161 pentapeptide, which has been shown to block against SARS-CoV-2 infection *in vivo* (Amruta et al., 2021)⁠. The RGD-based drug, Cilengitide, and the RGD peptide were also found to inhibit spike-integrins interactions on endothelial cells (Nader et al., 2021; Robles et al., 2021)⁠. Such studies highlight that inhibiting spike-integrin interactions may be useful in blocking or ameliorating infection. Gao et al. also suggested that administering entire integrin subunits could be helpful as a treatment option (Gao et al., 2021)⁠. Thus, additional work to define potential non-RGD-based integrin interactions may give rise to new drug target options. Experimental elucidation of the binding mechanisms and affinities between the SARS-CoV-2 spike protein and the diverse integrin dimers would be helpful for engineering inhibitory peptides or small molecules.

As ACE2 has been shown to associate with certain integrin subtypes, such as α_2_, α_5_ and β_1_, it is possible that integrin-binding motifs on the SARS-CoV-2 receptor-binding domain may be exposed even after binding with ACE2, which may then allow subsequent interaction between the spike-ACE2 complex and an integrin on the same receptor-binding domain (Clarke et al., 2012)⁠. However, since the coronavirus spike protein oligomerizes to its final trimeric form and, thus, contains three receptor-binding domains (one per protomer), the spike protein may interact with integrins in a variety of ways: the SARS-CoV-2 spike protein may 1) bind integrins independently of ACE2, 2) bind to ACE2 and then bind an integrin using another receptor-binding domain (or vice-versa) either on the same or a different spike protein, or 3) bind to ACE2, first, and then utilize accessible motifs to bind an integrin from the same spike receptor-binding domain (Makowski et al., 2021).

Evolutionary sequence comparisons between different coronavirus strains revealed that some of the putative integrin-binding motifs have recently emerged while others are more conserved among betacoronaviruses. Notably, all of the potential integrin-binding motifs, including the RGD sequence, were conserved in one pangolin coronavirus strain, which suggests that integrin-binding motifs may evolve before zoonotic transfer to humans. The presence of these motifs alongside the RGD motif on the pangolin coronavirus genome further supports their potential as mediators for integrin-binding. The discovery that the evolution of the RGD and other putative non-RGD integrin-binding motifs on the spike protein preludes the jump to humans reveals that the integrin-binding motifs may be a driving factor for adapting to human cells. These results indicate that the mapping of all known integrin-binding sequences to viral surface proteins may help to better understand the evolution of integrin use among viruses. Recorded mutations relevant to the putative integrin-binding motifs were centred around one motif similar to the ATN-161 pentapeptide, in particular, which might increase potential affinity to integrins. Monitoring future mutations on the spike protein receptor-binding domain may be useful to identify new cell entry receptors. Subsequent investigation into the connections between spontaneous integrin-binding motif generation and viral transmission between species may further delineate the role of integrins in zoonotic transfer.

The discovery of potential integrin-binding motifs independent of the RGD sequence on the SARS-CoV-2 spike protein highlights that integrin-binding motifs on viral surface proteins may be more widespread than previously established. Since integrins are ubiquitously expressed throughout the human body, their usage as receptors for cell entry by viruses should be scrutinized. The RGD and other putative integrin-binding motifs on the spike protein surface provide potential mechanisms through which SARS-CoV-2 may utilize integrins as cell entry receptors or to interfere with host signaling pathways. Further experimental work is necessary to validate the direct structural interactions between the SARS-CoV-2 spike protein and integrins.

## Data Availability Statement

The original contributions presented in the study are included in the article/**Supplementary Material**. Further inquiries can be directed to the corresponding author.

## Author Contributions

CB, SH, CL-HH, TB, and AJ all contributed to the conception and design of the study. CB performed sequence, structural, and statistical analyses. All authors contributed to the article and approved the submitted version.

## Funding

TB thanks the Wellcome Trust for support through an Investigator Award (200814/Z/16/Z; 2016 -2021). CB was supported by Antibiotic Research UK (ANTSRG 01/2019-PHZJ/687).

## Conflict of Interest

The authors declare that the research was conducted in the absence of any commercial or financial relationships that could be construed as a potential conflict of interest.

## Publisher’s Note

All claims expressed in this article are solely those of the authors and do not necessarily represent those of their affiliated organizations, or those of the publisher, the editors and the reviewers. Any product that may be evaluated in this article, or claim that may be made by its manufacturer, is not guaranteed or endorsed by the publisher.

## References

[B1] Aguirre C. Meca-Lallana V. Barrios-Blandino A. Del Río B. Vivancos J. (2020). Covid-19 in a Patient With Multiple Sclerosis Treated With Natalizumab: May the Blockade of Integrins Have a Protective Role? Mult. Scler. Relat. Disord. 44, 102250. doi: 10.1016/j.msard.2020.102250 32531754 PMC7831671

[B2] Amruta N. Engler-Chiurazzi E. B. Murray-Brown I. C. Gressett T. E. Biose I. J. Chastain W. H.. (2021). *In Vivo* Protection From SARS-CoV-2 Infection by ATN-161 in K18-Hace2 Transgenic Mice. Life Sci. 284, 119881. doi: 10.1016/j.lfs.2021.119881 34389403 PMC8352850

[B3] Aoki A. Adachi H. Mori Y. Ito M. Sato K. Okuda K.. (2021). A Rapid Screening Assay for L452R and T478K Spike Mutations in SARS-CoV-2 Delta Variant Using High-Resolution Melting Analysis. J. Toxicol. Sci. 46, 471–476. doi: 10.2131/jts.46.471 34602531

[B4] Armijos-Jaramillo V. Yeager J. Muslin C. Perez-Castillo Y. (2020). SARS-CoV-2, an Evolutionary Perspective of Interaction With Human ACE2 Reveals Undiscovered Amino Acids Necessary for Complex Stability. Evol. Appl. 13, 2168–2178. doi: 10.1111/eva.12980 PMC7262083 32837536

[B5] Barczyk M. Carracedo S. Gullberg D. (2010). Integrins. Cell Tissue Res. 339, 269–280. doi: 10.1007/s00441-009-0834-6 19693543 PMC2784866

[B6] Beaudoin C. A. Jamasb A. R. Alsulami A. F. Copoiu L. van Tonder A. J. Hala S.. (2021). Predicted Structural Mimicry of Spike Receptor-Binding Motifs From Highly Pathogenic Human Coronaviruses. Comput. Struct. Biotechnol. J. 19, 3938–3953. doi: 10.1016/j.csbj.2021.06.041 PMC8249111 34234921

[B7] Beddingfield B. J. Iwanaga N. Chapagain P. P. Zheng W. Roy C. J. Hu T. Y.. (2021). The Integrin Binding Peptide, ATN-161, as a Novel Therapy for SARS-CoV-2 Infection. JACC Basic Transl. Sci. 6, 1–8. doi: 10.1016/j.jacbts.2020.10.003 33102950 PMC7566794

[B8] Berrio A. Gartner V. Wray G. A. (2020). Positive Selection Within the Genomes of SARS-CoV-2 and Other Coronaviruses Independent of Impact on Protein Function. PeerJ 8, e10234–e10234. doi: 10.7717/peerj.10234 33088633 PMC7571416

[B9] Bongiovanni D. Klug M. Lazareva O. Weidlich S. Biasi M. Ursu S.. (2021). SARS-CoV-2 Infection Is Associated With a Pro-Thrombotic Platelet Phenotype. Cell Death Dis. 12, 50. doi: 10.1038/s41419-020-03333-9 33414384 PMC7790351

[B10] Calver J. Joseph C. John A. E. Organ L. Fainberg H. Porte J.. (2021). S31 The Novel Coronavirus SARS-CoV-2 Binds RGD Integrins and Upregulates Avb3 Integrins in Covid-19 Infected Lungs. Thorax 76, A22 LP–A22A23. doi: 10.1136/thorax-2020-BTSabstracts.37

[B11] Cianfrocca M. E. Kimmel K. A. Gallo J. Cardoso T. Brown M. M. Hudes G.. (2006). Phase 1 Trial of the Antiangiogenic Peptide ATN-161 (Ac-PHSCN-NH(2)), a Beta Integrin Antagonist, in Patients With Solid Tumours. Br. J. Cancer 94, 1621–1626. doi: 10.1038/sj.bjc.6603171 16705310 PMC2361324

[B12] Clarke N. E. Fisher M. J. Porter K. E. Lambert D. W. Turner A. J. (2012). Angiotensin Converting Enzyme (ACE) and ACE2 Bind Integrins and ACE2 Regulates Integrin Signalling. PloS One 7, e34747. doi: 10.1371/journal.pone.0034747 22523556 PMC3327712

[B13] Dehury B. Raina V. Misra N. Suar M. (2020). Effect of Mutation on Structure, Function and Dynamics of Receptor Binding Domain of Human SARS-CoV-2 With Host Cell Receptor ACE2: A Molecular Dynamics Simulations Study. Biomol. Struct. Dyn. 1–15. doi: 10.1080/07391102.2020.1802348 PMC7484587 32762417

[B14] Desai N. Neyaz A. Szabolcs A. Shih A. R. Chen J. H. Thapar V.. (2020). Temporal and Spatial Heterogeneity of Host Response to SARS-CoV-2 Pulmonary Infection. Nat. Commun. 11, 6319. doi: 10.1038/s41467-020-20139-7 33298930 PMC7725958

[B15] Duchrow T. Shtatland T. Guettler D. Pivovarov M. Kramer S. Weissleder R. (2009). Enhancing Navigation in Biomedical Databases by Community Voting and Database-Driven Text Classification. BMC Bioinf. 10, 317. doi: 10.1186/1471-2105-10-317 PMC2768718 19799796

[B16] Etzioni A. (1999). Integrins—the Glue of Life. Lancet 353, 341–343. doi: 10.1016/S0140-6736(05)74944-8 9950435

[B17] Fantini J. Yahi N. (2015). Chapter 12 - Viral and Bacterial Diseases. Eds. Fantini J. T.-B. L N. B. F S. Yahi N. D. (San Diego: Academic Press), 279–311. doi: 10.1016/B978-0-12-800111-0.00012-6

[B18] Futosi K. Fodor S. Mócsai A. (2013). Neutrophil Cell Surface Receptors and Their Intracellular Signal Transduction Pathways. Int. Immunopharmacol. 17, 638–650. doi: 10.1016/j.intimp.2013.06.034 23994464 PMC3827506

[B19] Gao S. Lu Y. Luan J. Zhang L. (2021). Low Incidence Rate of Diarrhoea in COVID-19 Patients Is Due to Integrin. J. Infect. 83, 496–522. doi: 10.1016/j.jinf.2021.07.007 PMC8280609 34274362

[B20] Gisby J. Clarke C. L. Medjeral-Thomas N. Malik T. H. Papadaki A. Mortimer P. M.. (2021). Longitudinal Proteomic Profiling of Dialysis Patients With COVID-19 Reveals Markers of Severity and Predictors of Death. Elife 10, e64827. doi: 10.7554/eLife.64827 33704068 PMC8064756

[B21] Greaney A. J. Starr T. N. Barnes C. O. Weisblum Y. Schmidt F. Caskey M.. (2021). Mapping Mutations to the SARS-CoV-2 RBD That Escape Binding by Different Classes of Antibodies. Nat. Commun. 12, (4196), 1–14. doi: 10.1038/s41467-021-24435-8 34234131 PMC8263750

[B22] Guruprasad L. (2021). Human SARS CoV-2 Spike Protein Mutations. Proteins Struct. Funct. Bioinforma. 89, 569–576. doi: 10.1002/prot.26042 PMC8014176 33423311

[B23] Hamaia S. W. Pugh N. Raynal N. Némoz B. Stone R. Gullberg D.. (2012). Mapping of Potent and Specific Binding Motifs, GLOGEN and GVOGEA, for Integrin α1β1 Using Collagen Toolkits II and III. J. Biol. Chem. 287, 26019–26028. doi: 10.1074/jbc.M112.353144 22654115 PMC3406685

[B24] Hussein H. A. M. Walker L. R. Abdel-Raouf U. M. Desouky S. A. Montasser A. K. M. Akula S. M. (2015). Beyond RGD: Virus Interactions With Integrins. Arch. Virol. 160, 2669–2681. doi: 10.1007/s00705-015-2579-8 26321473 PMC7086847

[B25] Jokinen J. White D. J. Salmela M. Huhtala M. Käpylä J. Sipilä K.. (2010). Molecular Mechanism of Alpha2beta1 Integrin Interaction With Human Echovirus 1. EMBO J. 29, 196–208. doi: 10.1038/emboj.2009.326 PMC2808374 19927126

[B26] Kabsch W. Sander C. (1983). Dictionary of Protein Secondary Structure: Pattern Recognition of Hydrogen-Bonded and Geometrical Features. Biopolymers 22, 2577–2637. doi: 10.1002/bip.360221211 6667333

[B27] Kliche J. Kuss H. Ali M. Ivarsson Y. (2021). Cytoplasmic Short Linear Motifs in ACE2 and Integrin β3 Link SARS-CoV-2 Host Cell Receptors to Mediators of Endocytosis and Autophagy. Sci. Signal 14, eabf1117. doi: 10.1126/scisignal.abf1117 33436498 PMC7928716

[B28] Kotani N. Nakano T. (2020). Candidate Screening of Host Cell Membrane Proteins Involved in SARS-CoV-2 Entry. bioRxiv. doi: 10.1101/2020.09.09.289488. 2020.09.09.289488.

[B29] Kumar M. Gouw M. Michael S. Sámano-Sánchez H. Pancsa R. Glavina J.. (2020). ELM-The Eukaryotic Linear Motif Resource in 2020. Nucleic Acids Res. 48, D296–D306. doi: 10.1093/nar/gkz1030 31680160 PMC7145657

[B30] Kuriata A. Gierut A. M. Oleniecki T. Ciemny M. P. Kolinski A. Kurcinski M.. (2018). CABS-Flex 2.0: A Web Server for Fast Simulations of Flexibility of Protein Structures. Nucleic Acids Res. 46, W338–W343. doi: 10.1093/nar/gky356 29762700 PMC6031000

[B31] Lanier L. L. (2008). Evolutionary Struggles Between NK Cells and Viruses. Nat. Rev. Immunol. 8, 259–268. doi: 10.1038/nri2276 18340344 PMC2584366

[B32] Letunic I. Bork P. (2021). Interactive Tree Of Life (iTOL) V5: An Online Tool for Phylogenetic Tree Display and Annotation. Nucleic Acids Res. 49, W293–W296. doi: 10.1093/nar/gkab301 33885785 PMC8265157

[B33] Li X. Giorgi E. E. Marichannegowda M. H. Foley B. Xiao C. Kong X.-P.. (2020). Emergence of SARS-CoV-2 Through Recombination and Strong Purifying Selection. Sci. Adv. 6, eabb9153. doi: 10.1126/sciadv.abb9153 32937441 PMC7458444

[B34] Liu P. Jiang J.-Z. Wan X.-F. Hua Y. Li L. Zhou J.. (2020). Are Pangolins the Intermediate Host of the 2019 Novel Coronavirus (SARS-CoV-2)? PloS Pathog. 16, e1008421. doi: 10.1371/journal.ppat.1008421 32407364 PMC7224457

[B35] Liu Q. Lusso P. (2020). Integrin α4β7 in HIV-1 Infection: A Critical Review. J. Leukoc. Biol. 108, 627–632. doi: 10.1002/JLB.4MR0120-208R 32272507

[B36] Luan J. Lu Y. Gao S. Zhang L. (2020). A Potential Inhibitory Role for Integrin in the Receptor Targeting of SARS-CoV-2. J. Infect. 81, 318–356. doi: 10.1016/j.jinf.2020.03.046 PMC7151354 32283163

[B37] Luo B.-H. Carman C. V. Springer T. A. (2007). Structural Basis of Integrin Regulation and Signaling. Annu. Rev. Immunol. 25, 619–647. doi: 10.1146/annurev.immunol.25.022106.141618 17201681 PMC1952532

[B38] Madeira F. Park Y. M. Lee J. Buso N. Gur T. Madhusoodanan N.. (2019). The EMBL-EBI Search and Sequence Analysis Tools APIs in 2019. Nucleic Acids Res. 47, W636–W641. doi: 10.1093/nar/gkz268 30976793 PMC6602479

[B39] Maginnis M. S. (2018). Virus-Receptor Interactions: The Key to Cellular Invasion. J. Mol. Biol. 430, 2590–2611. doi: 10.1016/j.jmb.2018.06.024 29924965 PMC6083867

[B40] Makarem R. Humphries M. J. (1991). LDV: A Novel Cell Adhesion Motif Recognized by the Integrin α4β1. Biochem. Soc Trans. 19, 380S–380S. doi: 10.1042/bst019380s 1724433

[B41] Makowski L. Olson-Sidford W. W-Weisel J. (2021). Biological and Clinical Consequences of Integrin Binding *via* a Rogue RGD Motif in the SARS CoV-2 Spike Protein. Viruses 13, 146. doi: 10.3390/v13020146 33498225 PMC7909284

[B42] Michel N. Allespach I. Venzke S. Fackler O. T. Keppler O. T. (2005). The Nef Protein of Human Immunodeficiency Virus Establishes Superinfection Immunity by a Dual Strategy to Downregulate Cell-Surface CCR5 and CD4. Curr. Biol. 15, 714–723. doi: 10.1016/j.cub.2005.02.058 15854903

[B43] Nader D. Fletcher N. Curley G. F. Kerrigan S. W. (2021). SARS-CoV-2 Uses Major Endothelial Integrin αvβ3 to Cause Vascular Dysregulation *In-Vitro* During COVID-19. PloS One 16, e0253347. doi: 10.1371/journal.pone.0253347 34161337 PMC8221465

[B44] Othman H. Messaoud H. Ben, Khamessi O. Mabrouk H. Ben, Ghedira K. Bharuthram A.. (2021). SARS-CoV-2 Spike Protein Unlikely to Bind to Integrins *via* the Arg-Gly-Asp (RGD) Motif of the Receptor Binding Domain: Evidence From Structural Analysis and Microscale Accelerated Molecular Dynamics. bioRxiv. doi: 10.1101/2021.05.24.445335. 2021.05.24.445335. PMC8883519 35237662

[B45] Park E. J. Myint P. K. Appiah M. G. Darkwah S. Caidengbate S. Ito A.. (2021). The Spike Glycoprotein of SARS-CoV-2 Binds to β1 Integrins Expressed on the Surface of Lung Epithelial Cells. Viruses 13, 645. doi: 10.3390/v13040645 33918599 PMC8069079

[B46] Price M. N. Dehal P. S. Arkin A. P. (2010). FastTree 2 – Approximately Maximum-Likelihood Trees for Large Alignments. PloS One 5, e9490. doi: 10.1371/journal.pone.0009490 20224823 PMC2835736

[B47] Robles J. P. Zamora M. de la Escalera G. M. Clapp C. (2021). The Spike Protein of SARS-CoV-2 Induces Endothelial Inflammation Through Integrin α5β1 and NF-κb. bioRxiv. doi: 10.1101/2021.08.01.454605. 2021.08.01.454605. PMC8820157 35143839

[B48] Roivainen M. Piirainen L. Hovi T. Virtanen I. Riikonen T. Heino J.. (1994). Entry of Coxsackievirus A9 Into Host Cells: Specific Interactions With Alpha V Beta 3 Integrin, the Vitronectin Receptor. Virology 203, 357–365. doi: 10.1006/viro.1994.1494 7519807

[B49] Ruoslahti E. (1996). RGD and Other Recognition Sequences for Integrins. Annu. Rev. Cell Dev. Biol. 12, 697–715. doi: 10.1146/annurev.cellbio.12.1.697 8970741

[B50] Schmidt K. Keller M. Bader B. L. Korytář T. Finke S. Ziegler U.. (2013). Integrins Modulate the Infection Efficiency of West Nile Virus Into Cells. J. Gen. Virol. 94, 1723–1733. doi: 10.1099/vir.0.052613-0 23658209 PMC3749529

[B51] Sebé-Pedrós A. Roger A. J. Lang F. B. King N. Ruiz-Trillo I. (2010). Ancient Origin of the Integrin-Mediated Adhesion and Signaling Machinery. Proc. Natl. Acad. Sci. 107, 10142 LP – 10147. doi: 10.1073/pnas.1002257107 20479219 PMC2890464

[B52] Shtatland T. Guettler D. Kossodo M. Pivovarov M. Weissleder R. (2007). PepBank–a Database of Peptides Based on Sequence Text Mining and Public Peptide Data Sources. BMC Bioinf. 8, 280. doi: 10.1186/1471-2105-8-280 PMC1976427 17678535

[B53] Simons P. Rinaldi D. A. Bondu V. Kell A. M. Bradfute S. Lidke D.. (2021). Integrin Activation Is an Essential Component of SARS-CoV-2 Infection. bioRxiv. 2021.07.20.453118. doi: 10.1101/2021.07.20.453118 PMC8516859 34650161

[B54] Spinello A. Saltalamacchia A. Magistrato A. (2020). Is the Rigidity of SARS-CoV-2 Spike Receptor-Binding Motif the Hallmark for Its Enhanced Infectivity? Insights From All-Atom Simulations. J. Phys. Chem. Lett. 11, 4785–4790. doi: 10.1021/acs.jpclett.0c01148 32463239

[B55] Spitaleri A. Mari S. Curnis F. Traversari C. Longhi R. Bordignon C.. (2008). Structural Basis for the Interaction of isoDGR With the RGD-Binding Site of αvβ3 Integrin. J. Biol. Chem. 283, 19757–19768. doi: 10.1074/jbc.M710273200 18480047

[B56] Takada Y. Ye X. Simon S. (2007). The Integrins. Genome Biol. 8, 215. doi: 10.1186/gb-2007-8-5-215 17543136 PMC1929136

[B57] Tresoldi I. Sangiuolo C. F. Manzari V. Modesti A. (2020). SARS-COV-2 and Infectivity: Possible Increase in Infectivity Associated to Integrin Motif Expression. J. Med. Virol. 92, 1741–1742. doi: 10.1002/jmv.25831 32246503 PMC7228266

[B58] Vanderheiden A. Ralfs P. Chirkova T. Upadhyay A. A. Zimmerman M. G. Bedoya S.. (2020). Type I and Type III Interferons Restrict SARS-CoV-2 Infection of Human Airway Epithelial Cultures. J. Virol. 94, e00985–20. doi: 10.1128/jvi.00985-20 PMC7495371 32699094

[B59] van Golen K. L. Bao L. Brewer G. J. Pienta K. J. Kamradt J. M. Livant D. L.. (2002). Suppression of Tumor Recurrence and Metastasis by a Combination of the PHSCN Sequence and the Antiangiogenic Compound Tetrathiomolybdate in Prostate Carcinoma. Neoplasia 4, 373–379. doi: 10.1038/sj.neo.7900258 12192595 PMC1564117

[B60] Wang C. Dinesh R. K. Qu Y. Rustagi A. Cousins H. Zengel J.. (2021). CRISPRa Screening With Real World Evidence Identifies Potassium Channels as Neuronal Entry Factors and Druggable Targets for SARS-CoV-2. bioRxiv. doi: 10.1101/2021.07.01.450475. 2021.07.01.450475.

[B61] Wise J. (2021). Covid-19: The E484K Mutation and the Risks It Poses. BMJ 372:n359. doi: 10.1136/bmj.n359 33547053

[B62] Wu M. Chen Y. Xia H. Wang C. Tan C. Y. Cai X.. (2020). Transcriptional and Proteomic Insights Into the Host Response in Fatal COVID-19 Cases. Proc. Natl. Acad. Sci. U. S. A. 117, 28336–28343. doi: 10.1073/pnas.2018030117 33082228 PMC7668053

[B63] Xiong J.-P. Stehle T. Zhang R. Joachimiak A. Frech M. Goodman S. L.. (2002). Crystal Structure of the Extracellular Segment of Integrin αvβ3 in Complex With an Arg-Gly-Asp Ligand. Science (80-). 296, 151 LP – 155. doi: 10.1126/science.1069040 11884718

[B64] Zamorano Cuervo N. Grandvaux N. (2020). ACE2: Evidence of Role as Entry Receptor for SARS-CoV-2 and Implications in Comorbidities. Elife 9, e61390. doi: 10.7554/eLife.61390 33164751 PMC7652413

[B65] Zhang J. Wang J. Gao N. Chen Z. Tian Y. An J. (2007). Up-Regulated Expression of β3 Integrin Induced by Dengue Virus Serotype 2 Infection Associated With Virus Entry Into Human Dermal Microvascular Endothelial Cells. Biochem. Biophys. Res. Commun. 356, 763–768. doi: 10.1016/j.bbrc.2007.03.051 17382900

